# The impact of trait mindfulness on college students' exercise commitment: a chain mediation model

**DOI:** 10.3389/fpsyg.2026.1753741

**Published:** 2026-08-14

**Authors:** Li Chuang, Zhao Cheng Bo, Zhou Wei Dong, Zhu Ran

**Affiliations:** 1 Basic Teaching Department, Henan Polytechnic, Henan, Zhengzhou, China; 2 Faculty of Philosophy and Political Science, Al-Farabi Kazakh National University, Almaty, Kazakhstan; 3 School of Arts and Sports, Henan Open University, Henan, Zhengzhou, China; 4 Department of Physical Education and Research, Henan Logistics Vocational College, Henan, Zhengzhou, China

**Keywords:** exercise commitment, exercise motivation, physical activity, positive emotions, trait mindfulness

## Abstract

**Objective:**

As a pivotal demographic in society, college students play a key role in shaping future social development. Investigating exercise motivation, positive emotions, and physical exercise commitment is crucial for enabling timely identification of issues, enhancing their enthusiasm for physical activity, and providing both theoretical and practical insights to foster motivation, improve affective experiences, and increase commitment in exercise.

**Methods:**

Using cluster sampling, 504 college students from five universities in Henan Province were surveyed. A chain mediation model was constructed, with exercise motivation and positive emotions as sequential mediators, to explore the underlying mechanisms linking trait mindfulness to exercise commitment.

**Results:**

Significant differences in exercise commitment were observed across gender, major, and levels of trait mindfulness (*p* < 0.01). Trait mindfulness, exercise commitment, exercise motivation, and positive emotions were all significantly correlated with each other (p <0.01). Trait mindfulness significantly and positively predicted exercise commitment (β = 0.34, *t* = 8.43, *p* < 0.001), exercise motivation (β = 0.31, *t* = 7.18, *p* < 0.001), and positive emotions (β = 0.23, *t* = 5.39, *p* < 0.001). The indirect effect of trait mindfulness on exercise commitment through the sequential path of exercise motivation and positive emotions was significant, with a 95% confidence interval of [0.014, 0.067].

**Conclusion:**

Exercise motivation and positive emotions exert a significant chain mediation effect between trait mindfulness and exercise commitment. While trait mindfulness serves as a foundational factor, exercise motivation and affective experiences during physical activity are the key proximal drivers.

## 1 Introduction

With the rapid development of internet technology and the digital economy, online games and short videos have increasingly consumed much of college students' leisure time. As a result, their participation in physical activity has markedly declined, their exercise motivation has become notably insufficient, and their level of exercise commitment remains low—contributing to an overall deterioration in their physical health. Given their role as future leaders, understanding college students' exercise behavior is of particular importance. Investigating their exercise motivation, positive emotions, and exercise commitment can deepen our understanding of their exercise behaviors, facilitate timely identification of underlying issues, and enhance their enthusiasm for physical activity. This study constructs a theoretical model based on Self-Determination Theory (SDT) (Ryan and Deci, 2024) and Broaden-and-Build Theory (Liangshi, 2025) to integrate the relationship between trait mindfulness, exercise motivation, positive emotions, and exercise commitment. First, according to SDT theory, an individual's behavioral persistence depends on the quality of motivation, and autonomous motivation is key to maintaining long-term behavioral engagement (Stone et al., 2009). Trait mindfulness, as a non-judgmental present awareness, helps individuals perceive their inner needs more clearly and reduces interference from external pressures, thereby promoting the formation of autonomous motivation. Second, Broaden-and-Build Theory points out that positive emotions can expand an individual's instantaneous thought-action scope and construct lasting personal resources. In the context of physical exercise, the satisfaction of exercise motivation triggers positive emotional experiences, which in turn enhance an individual's vitality and focus, promoting a higher level of exercise engagement. In summary, this study combines the motivational mechanism of SDT with the emotional mechanism of Expansion-Construction Theory, aiming to reveal the internal mechanism by which trait mindfulness influences college students' exercise engagement from an integrated perspective of “cognition-motivation-emotion-behavior”. Therefore, this study examines the relationships among trait mindfulness, exercise motivation, positive emotions, and exercise commitment among college students, offering both theoretical insights and practical implications for fostering exercise motivation, improving affective experiences during physical activity, and increasing behavioral engagement.

### 1.1 The relationship between trait mindfulness and exercise commitment

Mindfulness is defined as a state of mind in which an individual focuses their attention on the external environment and internal psychology that are happening in the present moment (Dane, 2011). Trait mindfulness is a psychological quality that an individual is born with and does not require mindfulness training (Wei, 2020). It is the ability to focus on present experience (Brown and Ryan, 2003). Researchers found that trait mindfulness has a positive effect on work engagement (Yu et al., 2022; Leroy et al., 2013). Its impact on work engagement is mainly through the following aspects: First, the core feature of trait mindfulness is to focus attention steadily and continuously on what is being done, so that individuals can maintain a state of concentration (Wadlinger and Isaacowitz, 2011). In a state of total absorption, a person is fully engaged and involved in a particular role, activity, or task (Agarwal and Karahanna, 2000). Secondly, by enhancing self-awareness of emotions, thoughts, etc., more autonomous motivation is exercised, thus supporting work commitment (Brown et al., 2007). Finally, by providing self-regulatory functions that draw attention and awareness to current experiences, people can stay more focused (Glomb et al., 2011). Since the concept of physical exercise investment is adapted from other fields such as work and study, it is integrated and differentiated based on the commonalities and characteristics of physical exercise, work and study (Dong, 2017). The promoting effect of trait mindfulness on work commitment (Malinowski and Lim, 2015). The same applies to the promotion of physical exercise investment. Therefore, this study proposes the hypothesis that trait mindfulness positively predicts college students' Physical exercise investment.

### 1.2 The mediating role of exercise motivation

Motivation is the psychological drive that energizes and directs an individual's behavior (Zhang and Mao, 2003). Goal-oriented functionality, In addition, motivation also has the function of directing and stimulating behaviors (Peng, 2012). Exercise motivation is the internal psychological activity of an individual when participating in physical exercise activities. It promotes the individual to actively participate in physical exercise activities under the guidance of exercise goals. It is the internal demand for physical exercise generated by the interaction of incentives such as the exercise environment and physical exercise goals (Yang, 2016). Exercise motivation is affected by many factors. Some studies have shown that exercise motivation is affected by gender, age and education level (Xu, 2020). The influence of social support, self-efficacy, etc.; the current field of mindfulness also involves the study of exercise motivation. Personality traits, as a kind of psychological resource, are unique and stable psychological qualities of an individual. They can control individual behavior and predict behavioral motivation. They are an important factor in judging the strength of exercise motivation and play a guiding role in exercise motivation (Wei, 2020). Zhu Congqing et al. pointed out that personality traits have a significant positive impact on exercise motivation (Zhu and Dong, 2016). Trait mindfulness is highly correlated with personality traits, so it has a significant impact on exercise motivation (Wei, 2020; Kang et al., 2017). The above analysis shows that trait mindfulness has an important impact on exercise motivation.

Learning commitment is a state in which students show a continuous and positive attitude toward learning during the learning process (Yang et al., 2021). Studies have found that learning motivation is the basis of learning investment (Ji and Wang, 2016). It is also an important predictor of learning investment (Dierendonck et al., 2021; Wilkie and Sullivan, 2018). It is the source of motivation for students to learn (Ryan and Deci, 2020). Behavior is driven by various motivations, and learning input as a behavioral manifestation of learning is driven by learning motivation (Yin and Wang, 2016). The stronger the individual's motivation is when learning. The more motivation one has for learning activities, the higher the level of involvement one can show. Therefore, the direct influencing factor of learning involvement behavior is learning motivation (Dong, 2021). Physical exercise is also a form of learning behavior, and the relationship between learning motivation and learning investment also applies to exercise motivation and exercise investment. Studies have shown that there is a close connection between exercise motivation and Physical exercise investment. Clear exercise motivation helps strengthen individuals' understanding of the value of exercise, enhance their effort and concentration during exercise, and improve their state of Physical exercise investment (Li and Li, 2020). Therefore, based on the relationships between trait mindfulness and exercise motivation and between exercise motivation and Physical exercise investment, we hypothesized that exercise motivation mediates the relationship between trait mindfulness and Physical exercise investment.

### 1.3 The mediating role of positive emotions

Positive emotions are immediate and unique reactions to things that a person finds meaningful (Fredrickson, 2001). It is associated with the satisfaction of certain needs, accompanied by a pleasant subjective experience, and improves people's enthusiasm and ability to move (Meng, 2005). Studies have shown that college students' emotional regulation methods can significantly affect their exercise motivation. Cognitive reappraisal and exercise motivation have reached a moderate positive correlation. College students who tend to cognitive reappraisal experience more positive emotions and have stronger exercise motivation (Zhu and Dong, 2016). The study found that college students with high emotional intelligence have a higher level of self-efficacy, which enables individuals to devote themselves to activities and tasks with higher emotions. This is because people with high emotional intelligence can deal with the pressure they face with a positive and optimistic attitude. When facing pressure, they can subjectively examine their own emotions and actively deal with problems, which has a positive significance for improving work commitment (Zhu et al., 2016). Positive emotions can expand an individual's cognitive scope (Li, 2018). Cognitive reappraisal can also better regulate emotions. Cognitive reappraisal has a positive impact on Physical exercise investment. Neuropsychology believes that cognitive reappraisal is conducive to guiding behavioral cognition and stimulating behavioral vitality. If college students perceive that physical exercise has the value of promoting health and enhancing friendship during exercise, they will show a positive, immersed, and focused state of commitment during exercise (Zhu et al., 2016). In addition, studies have shown that trait mindfulness and exercise motivation have a significant impact on positive emotions. Positive emotions may influence the relationship between trait mindfulness and exercise motivation, trait mindfulness and Physical exercise investment, and exercise motivation and Physical exercise investment. Some studies have also found that positive emotions play a mediating role in the relationship between other variables. Therefore, this study hypothesizes that positive emotions may play a mediating role in the relationship between trait mindfulness and exercise commitment, and between exercise motivation and exercise commitment.

**Figure 1:**
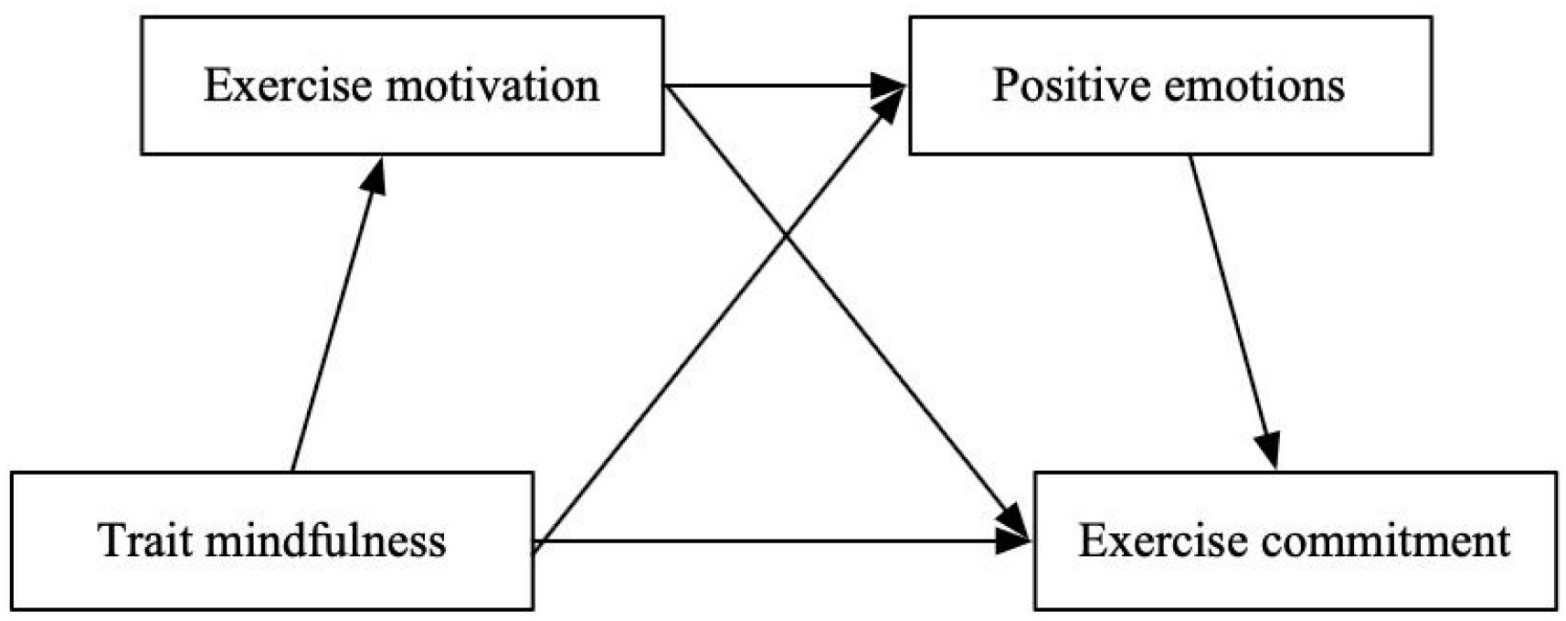
Theoretical model.

In summary, after comprehensively analyzing the relationship between the four variables of trait mindfulness, exercise motivation, Physical exercise investment, and positive emotions, this study constructed a chain mediation model (see Figure 1) to examine the mediating mechanism of exercise motivation and positive emotions in the prediction of college students' physical exercise investment by trait mindfulness, in order to clarify the cognitive mechanism and individual differences of trait mindfulness on college students' physical exercise motivation and Physical exercise investment, and provide a reference for enriching and deepening the research on exercise motivation and Physical exercise investment.

## 2 Research objects and methods

### 2.1 Research subjects

This study adhered to the ethical guidelines of the Declaration of Helsinki and was approved by the Biomedical Research Ethics Subcommittee of Henan University (No.: HUSONM2025-979). All participants signed the informed consent form before completing the questionnaire and were informed that the study was completely anonymous, the data would be used only for academic research, and they had the right to withdraw from the survey at any time. During the administration of the questionnaire, graduate students majoring in psychology who had received standardized training served as the proctors, and uniformly read the instructions to the students, emphasizing the independence and authenticity of the questionnaire completion. Cluster sampling was employed in this study, using the natural administrative classes of universities as the basic sampling unit. The specific implementation steps are as follows: First, based on the geographical distribution and type of universities in Henan Province, five universities were randomly selected as sample schools. Second, within the selected universities, administrative class directories were obtained in cooperation with relevant departments, and several complete administrative classes were randomly selected from each grade using a random number table method to form the survey group. Finally, cluster testing was conducted on the selected classes. This study conducted an online survey using the Wenjuanxing platform. Although the platform mandated core questions and required completion before submission, it only restricted mandatory content; demographic data and some supplementary questions were optional, potentially leading to missing data. Furthermore, the platform could not assess the quality of responses, resulting in instances of perfunctory answers such as excessively short response times, repetitive answer patterns, and logical inconsistencies. A total of 567 questionnaires were collected. Following academic research standards, invalid questionnaires with numerous missing values or careless responses were removed, ultimately confirming 504 valid questionnaires, including 265 from males and 239 from females.

### 2.2 Measurement tools

#### 2.2.1 Trait mindfulness

Mindful Attention Awareness Scale developed by Berown and Ryan and revised by Deng et al. was used for the assessment. This scale consists of 15 items, each scored from 1 to 6, using a 6-point scale. All items are reverse-scored and summed to calculate a trait mindfulness score. An average score of 4.5 or higher indicates high trait mindfulness, 1.5–4.5 indicates moderate trait mindfulness, and below 1.5 indicates low trait mindfulness. The Cronbach's α coefficient for this scale was 0.82.

#### 2.2.2 Exercise motivation

The simplified version of the Exercise Motivation Scale (MPAM-R), revised by Chen et al. (2013) was used. This scale consists of five dimensions: social motivation, enjoyment motivation, health motivation, appearance motivation, and ability motivation. It contains 15 items, each scored on a 5-point Likert scale: “strongly disagree,” “somewhat disagree,” “neither agree nor disagree,” “somewhat agree,” and “strongly agree.” Exercise motivation levels were determined based on the average score: an average score of 3.75 or higher indicates high motivation, 1.25–3.75 indicates moderate motivation, and below 1.25 indicates low motivation. The Cronbach's α coefficient for this scale was 0.917.

#### 2.2.3 Positive emotions

The positive emotion subscale of the Positive and Negative Emotion Scale, revised by Huang et al. (2003) was used. The scale includes items related to positive emotions, such as being interested, excited, strong, and enthusiastic. A five-point Likert scale was used, with “1–5” representing the degree of activation, from low to high. Higher scores indicate higher levels of positive emotion.

#### 2.2.4 Exercise commitment

The self-rating scale for college students' physical exercise commitment, developed by Dong (2017), was used. It is divided into four dimensions: vitality and persistence, focus and satisfaction, participation and autonomy, and value perception. Each dimension contains five items, for a total of 20 items, using a five-point scoring system. The Cronbach's α coefficient for this scale was 0.89.

#### 2.2.5 Confirmatory factor analysis (CFA)

The data in this study were self-reported scale data from the participants. A common method factor test based on the CFA was used to control for common method bias. The process and results are as follows: First, the original multivariate model was constructed, and the fit results showed that the model fit was good (χ^2^ = 802.36, df = 629, *p* < 0.001; *RMSEA* = 0.035, *CFI* = 0.952, *TLI* = 0.947, *SRMR* = 0.051). Then, a global common method factor was added, loading all items simultaneously onto both the theoretical and method factors. The fit index was (χ^2^ = 793.10, df = 628, *p* < 0.001; *RMSEA* = 0.034, *CFI* = 0.953, *TLI* = 0.948, *SRMR* = 0.050). Finally, model comparison revealed only minor fluctuations in the core fit indices, with minimal chi-square differences. The effect size was Δχ^2^ = 9.26 (Δdf = 1, *p* = 0.002) and the effect size was very small, indicating that there was no serious common method bias in this study.

### 2.3 Statistical analysis

This study primarily used SPSS to conduct statistical analysis of the data, and SPSS and PROCESS were used to test the effect of the mediation model.

### 2.4 Data collection procedures and quality control

To ensure the rigor and reproducibility of the data, this study implemented a rigorous quality control procedure: First, response time was monitored, with the system backend recording the response time for each questionnaire. Questionnaires with a response time of less than 120 s were discarded to rule out random completion. Second, pattern recognition was performed, checking for obvious patterns in responses (such as selecting the same option for more than 10 consecutive questions); such questionnaires were considered invalid.

## 3 Results

### 3.1 Common method bias test

An exploratory factor analysis was conducted on the four dimensional measurement items using the Harman single-factor test to examine common method bias. A single-factor test was performed on the collected data. Eight factors were found to have eigenvalues greater than 1, and the first factor explained 25.32% of the variance, which is lower than the critical value of 40%, indicating that there was no significant common method bias in this study.

### 3.2 Correlation analysis of variables

Correlation analysis was conducted among college students' trait mindfulness, exercise motivation, Physical exercise investment, and positive emotions (see Table 1). The results showed that trait mindfulness was significantly correlated with exercise motivation, Physical exercise investment, and positive emotions; exercise motivation was significantly positively correlated with Physical exercise investment and positive emotions; and Physical exercise investment was significantly positively correlated with positive emotions. These intervariate correlations provided a basis for subsequent mediation effect testing, indicating that exercise motivation and positive emotions may mediate the relationship between trait mindfulness and Physical exercise investment (see Table 1).

**Table 1:** Standard deviation of total score, total score mean and related relationships.

Variable	*M* ±*SD*	Trait mindfulness	Exercise motivation	Exercise commitment	Positive emotions
Trait mindfulness	63.75 ± 12.956	1			
Exercise motivation	64.88 ± 10.321	0.308^**^	1		
Exercise commitment	74.3 ± 14.584	0.310^**^	0.387^***^	1	
Positive emotions	34.93 ± 6.853	0.289^**^	0.313^**^	0.635^***^	1

^**^p < 0.01, ^***^p < 0.001, same below.

### 3.3 Testing the mediation model between exercise motivation and positive emotions

This study controlled for gender and profession in the mediation model based on the following: First, gender is the most stable demographic predictor in sports participation and psychosocial adaptation studies, and multinomial meta-analysis shows that it has a cross-cultural consistent impact on sports motivation and social interaction patterns (Hu et al., 2025; Li et al., 2025). Second, professional background (especially sports-related majors) directly influences individuals' cognition and practice of functional sports, and is a more substantial variable than grade level (Xue et al., 2023). Third, following the core principles of variable selection, we avoided controlling for variables that might be located along the mediation path (such as grade level and sports experience) to prevent over-control from leading to biased estimation of the mediation effect (Yu et al., 2025; Li et al., 2025). The selection of gender and major as control variables aligns with mainstream research in sports psychology and inclusive education, prioritizing these two factors. This choice ensures model simplicity while avoiding over-control.

**Figure 2:**
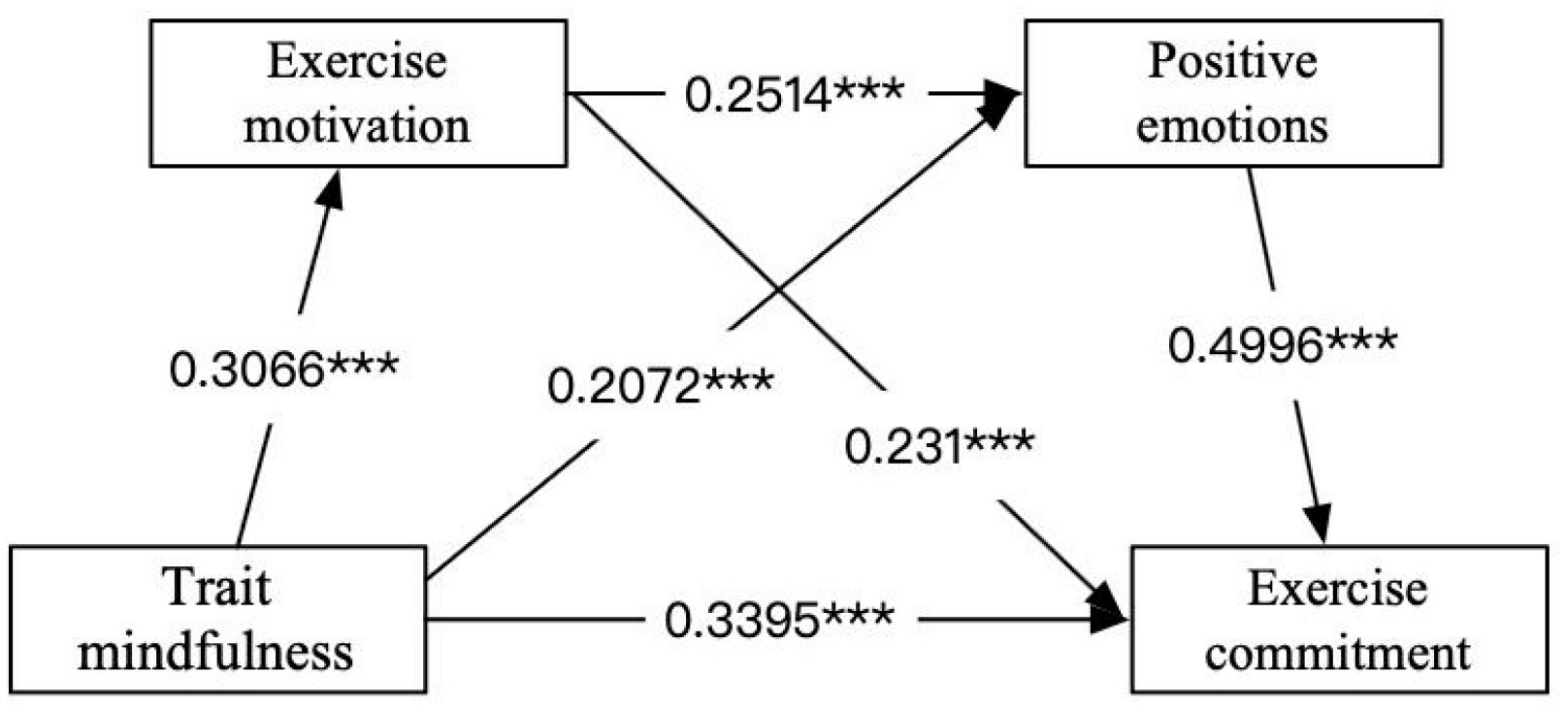
Chain mediation model diagram. ^***^p < 0.001.

Using the PROCESS plug-in in SPSS, model 6 was selected to test the mediating effect relationship between trait mindfulness, exercise motivation, Physical exercise investment and positive emotions while controlling for gender and major. The results (Table 2 and Figure 2) showed that, in the absence of a mediating variable, trait mindfulness had a significant positive predictive effect on Physical exercise investment (β = 0.34, *t* = 8.43, *p* < 0.01). Trait mindfulness also had a significant positive predictive effect on both exercise motivation (β = 0.31, *t* = 7.18, *p* < 0.01) and positive affect (β = 0.23, *t* = 5.39, *p* < 0.01), and exercise motivation also had a significant positive predictive effect on positive affect (β = 0.25, *t* = 5.88, *p* < 0.01). When exercise motivation and positive affect were added as mediating variables between trait mindfulness and Physical exercise investment, the positive predictive effects of exercise motivation (β = 0.21, *t* = 6.03, *p* < 0.01) and positive affect (β = 0.50, *t* = 14.36, *p* < 0.01) on Physical exercise investment became even more significant, preliminarily confirming the mediating role of exercise motivation and positive affect.

**Table 2:** Results of the chain mediation model.

Dependent variable	Independent variable	*R*	*F*	β	*t*	*p*
Exercise commitment		0.4443	40.9881			
	Gender			−0.2395	−5.9338	0
	Major			0.1947	4.8385	0
	Trait mindfulness			0.3395	8.4315	0
Exercise motivation		0.3112	17.8724			
	Gender			−0.0001	−0.0018	0.9985
	Major			−0.0412	−0.9658	0.3346
	Trait mindfulness			0.3066	7.1771	0
Positive emotions		0.419	26.5606			
	Gender			−0.1627	−3.9719	0.0001
	Major			0.09	2.2031	0.028
	Trait mindfulness			0.231	5.3852	0
	Exercise motivation			0.2514	5.8772	0
Exercise commitment		0.7093	100.8704			
	Gender			−0.1582	−4.8956	0
	Major			0.1634	5.1225	0
	Exercise motivation			0.2072	6.0277	0
	Positive emotions			0.4996	14.3622	0
	Trait mindfulness			0.1221	3.5594	0.0004

All variables in the model are entered using standardized data.

### 3.4 Verification of the mediation effect

Bootstrap was used to test the mediation path. The results are shown in Table 3. The 95% confidence interval for the path from trait mindfulness to exercise motivation to Physical exercise investment was 0.032 at the lower limit and 0.11 at the upper limit, which did not include 0, confirming that exercise motivation mediates the relationship between trait mindfulness and Physical exercise investment. The 95% confidence interval for the path from trait mindfulness to positive emotion to Physical exercise investment was 0.048 at the lower limit and 0.18 at the upper limit, which did not include 0, confirming that positive emotion mediates the relationship between trait mindfulness and Physical exercise investment. The 95% confidence interval for the path from trait mindfulness to exercise motivation to positive emotion to Physical exercise investment was 0.014 at the lower limit and 0.067 at the upper limit, which did not include 0, indicating that exercise motivation and positive emotion mediate the relationship between trait mindfulness and Physical exercise investment.

**Table 3:** Bootstrap analysis of significance test of mediation effect.

Impact path	Effect size	Boot	Boot *CI*	Boot *CI*	Effect ratio
		Standard error	Lower limit	Upper limit	
Trait mindfulness → exercise commitment	0.1221	0.0343	0.0547	0.1894	35.96%
Trait mindfulness → exercise motivation → exercise commitment	0.0635	0.0196	0.0318	0.108	18.71%
Trait mindfulness → positive emotions → exercise commitment	0.1154	0.0341	0.0481	0.182	33.99%
Trait mindfulness → exercise motivation → positive emotions → exercise commitment	0.0385	0.0136	0.0135	0.0667	11.34%
Total effect	0.3395	0.0403	0.2604	0.4186	100.00%

## 4 Analysis and discussion

### 4.1 The relationship between trait mindfulness and physical exercise investment among college students

This study found a significant positive correlation between trait mindfulness and college students' Physical exercise investment, indicating that trait mindfulness is an important factor affecting college students' physical exercise investment, verifying the research hypothesis. This is consistent with previous research results that trait mindfulness has a positive effect on commitment (Malinowski and Lim, 2015). The impact of trait mindfulness on commitment is mainly through self-regulation, Encourage students to regulate their thoughts and behaviors (Hammill et al., 2023), which keeps attention stable and continuously focused on what is being done at the moment, reduces wandering thoughts and the distribution of attention, and enables individuals to maintain a state of concentration (Wadlinger and Isaacowitz, 2011; Glomb et al., 2011). In a state of total focus, an individual is fully engaged and involved in a particular role, activity, or task (Yu et al., 2022). In addition, self-regulation function will reduce the individual's negative evaluation of challenging events, promote more positive emotions, be less affected by adverse events, and the individual can devote more energy to physical exercise (Zhang et al., 2018)].

### 4.2 The mediating role of exercise motivation

Exploring the mediating role of exercise motivation in the relationship between trait mindfulness and college students' Physical exercise investment not only helps to reveal the factors through which trait mindfulness has a positive impact on individual physical exercise from the perspective of cognitive processing, but also helps us understand the cognitive mechanism of college students' Physical exercise investment. This study found that trait mindfulness can predict college students' physical exercise investment through the mediating effect of exercise motivation, verifying the research hypothesis of this article.

On the one hand, a higher level of trait mindfulness helps to stimulate students' exercise motivation. The higher the level of students' trait mindfulness, the stronger their exercise motivation. This is consistent with the research results of Dong H. (2021) and Kang et al. (2017). This is because individuals with high trait mindfulness often show characteristics such as optimism, enthusiasm, and good communication skills, and have faith in doing things and are willing to help others. These traits will encourage individuals to guide exercise orientation in order to achieve different exercise goals, and have clear exercise cognition and strong self-control ability of exercise behavior, showing strong enough exercise motivation (Zhu and Dong, 2016). Second, individuals with higher levels of trait mindfulness experience lower negative emotions when exercising, and reduced negative emotions predict increased exercise motivation (Kang et al., 2017). On the other hand, if students have a clear motivation to exercise, they will work harder and focus more during the exercise, and can effectively avoid interference from external things (Fredrickson, 2001). This is because exercise motivation is directional, which helps students narrow the scope of their attention during exercise, increase their attention during exercise, and enable them to devote more mental activities to physical exercise (Yang, 2016). Skinner's reinforcement theory of motivation suggests that positive reinforcement can have a positive impact on students' commitment. For example, positive reinforcement, such as praise from teachers and peers, attention, and recognition from classmates, can generate strong motivation and lead to sustained commitment. Therefore, trait mindfulness can promote college students' commitment in exercise through positive exercise motivation.

### 4.3 The mediating role of positive emotions

The study found that trait mindfulness can significantly and positively predict positive emotions, and positive emotions can also significantly and positively predict Physical exercise investment. Positive emotions play a complete mediating role between trait mindfulness and Physical exercise investment, proving that the higher the positive emotions that high-trait mindfulness college students obtain during physical exercise, the more conducive it is to college students' commitment in physical exercise, verifying the research hypothesis of this article.

First, individuals with higher levels of mindfulness have stronger psychological resilience, which can reduce and eliminate negative emotions and enhance positive emotions (Foureur et al., 2013). Sustained positive emotions can increase a person's coping resources, resulting in relatively high cognitive flexibility, attention, and cognition. In addition, individuals with higher levels of mindfulness are more tolerant and peaceful, and can resolve negative emotions in a timely manner and better accept current experiences, which helps individuals release stress and better adapt to adversity (Vich, 2015). This will enhance the individual's ability to cope with stress. Acceptance of stress and an open mind are effective ways to stimulate individual psychology (Yu et al., 2022) and can generate higher positive emotions toward physical exercise. On the other hand, positive emotions can expand an individual's cognitive scope sequence, enabling the individual to break through the original constraints of thinking and expand the individual's attention scope (Li, 2016); it has a certain expansion effect on cognition, among which cognitive reappraisal has a significant positive impact on physical exercise investment. College students who use cognitive reappraisal more often have more positive emotional experiences with physical exercise and are more energetic and enthusiastic about exercise. The more positive emotions college students gain from physical exercise, the more they can correctly deal with the stress caused by exercise, and thus ensure that they focus on physical exercise activities.

### 4.4 Chain mediating effects of exercise motivation and positive emotions

Trait mindfulness has a significant direct positive predictive effect on college students' Physical exercise investment. After adding exercise motivation and positive emotions, the impact of trait mindfulness on Physical exercise investment is even more significant. This shows that exercise motivation and positive emotions have a complete chain mediating effect between trait mindfulness and Physical exercise investment. This is consistent with the results of previous studies. During physical exercise activities, the level of mindfulness can regulate exercise motivation (Ruffault et al., 2016) and emotional regulation (Zhu and Dong, 2016), enabling college students to be more proactive during physical exercise. It is worth noting that exercise motivation has a significant positive prediction effect on positive emotions, indicating that the higher the college students' motivation for physical exercise, the higher the positive exercise emotions they gain during exercise activities, and the higher their investment in physical exercise activities. The chain-mediation effect results of this study indicate that trait mindfulness not only directly affects exercise commitment but also indirectly influences it through a dual “motivation-emotion” pathway. This finding has significant theoretical implications. First, it validates the applicability of SDT in explaining exercise behavior, namely, that the “awareness” function provided by mindfulness is a key prerequisite for stimulating intrinsic motivation. Second, it reveals the bridging role of the “constructive” function of positive emotions in maintaining behavior. Specifically, the increased motivation brought about by mindfulness does not directly translate into behavior but requires the “conversion” and “amplification” of positive emotions to ultimately manifest as high levels of focus, energy, and dedication (exercise commitment). This finding fills the gap in previous studies that neglected the “emotional mediation mechanism”. The results of the chain mediation study show that trait mindfulness can regulate college students' motivation for physical exercise and thus affect their positive emotions toward exercise, while positive emotions can fully influence their commitment in exercise, proving that exercise motivation and positive emotions are the bridges connecting trait mindfulness and Physical exercise investment. The results of this study can accurately and clearly understand the relevant factors and internal mechanisms of the impact of trait mindfulness on Physical exercise investment. We should give full play to the chain mediating role of exercise motivation and positive emotions between trait mindfulness and Physical exercise investment, so that college students can participate in physical exercise activities more actively and proactively, thereby improving their physical fitness.

### 4.5 Limitations and future prospects

While this study revealed the underlying mechanism by which trait mindfulness influences exercise engagement, there are still limitations that need to be addressed in future research. Firstly, regarding the research design, this study employed a cross-sectional design, which only reveals the correlation between variables. Future studies could employ longitudinal tracking or experimental intervention designs (such as mindfulness intervention experiments) to further verify the causal relationships between variables. Secondly, regarding sample representativeness, the sample in this study mainly came from universities in Henan Province. Future research could expand to universities of different regions and types across the country to improve the generalizability of the conclusions. Thirdly, regarding measurement methods, future studies could introduce objective physiological indicators (such as heart rate variability) or multi-source assessments (such as teacher evaluations) to reduce the bias caused by relying solely on self-report methods.

## 5 Conclusion

The results of this study show: Trait mindfulness can directly and significantly positively affect college students' Physical exercise investment. After adding the two mediating variables of exercise motivation and positive emotions, the direct effect of trait mindfulness on Physical exercise investment decreased. Trait mindfulness has a significant positive predictive effect on exercise motivation, which can significantly predict college students' Physical exercise investment; exercise motivation plays a mediating role between trait mindfulness and Physical exercise investment. Trait mindfulness has a significant positive predictive effect on positive emotions, which can significantly predict college students' Physical exercise investment; positive emotions play a mediating role between trait mindfulness and Physical exercise investment. Exercise motivation and positive emotions have a chain mediation effect between trait mindfulness and college students' Physical exercise investment.

## Data Availability

The original contributions presented in the study are included in the article/supplementary material, further inquiries can be directed to the corresponding author.
